# Fanconi–Bickel syndrome in a Ugandan child – diagnostic challenges in resource-limited settings: a case report

**DOI:** 10.1186/s13256-020-02488-5

**Published:** 2020-09-30

**Authors:** Thereza Piloya, Hawa Ssematala, Lydia Paparu Dramani, Oliva Nalikka, Miriam Baluka, Victor Musiime

**Affiliations:** Department of Paediatrics, Makerere University College of Health Sciences, Kampala, Uganda

**Keywords:** Fanconi–Bickel syndrome, Children, Uganda

## Abstract

**Background:**

Fanconi–Bickel syndrome is an autosomal recessive disorder of glucose metabolism. It is an extremely rare disorder. Most cases have been reported in consanguineous communities. None of the cases have been reported in Black Africans in sub-Saharan Africa. This case was diagnosed 3 years after initial presentation due to diagnostic challenges and limited awareness of similar metabolic syndromes in our setting.

**Case presentation:**

We report the case of a 4-year-old boy, born to non-consanguineous Black African parents, who presented with failure to thrive and rachitic features in infancy. Clinical, laboratory, and radiological features were indicative of Fanconi–Bickel syndrome. No genetic testing was done. The diagnosis was made 3 years after the initial presentation due to diagnostic challenges. He showed clinical improvement with the institution of a galactose-free diet.

**Conclusion:**

Fanconi–Bickel syndrome occurs even in non-consanguineous Black African populations. Therefore, clinicians in resource-poor settings should raise their index of suspicion for such metabolic disorders in settings with a high prevalence of failure to thrive among children.

## Introduction

Fanconi–Bickel syndrome (FBS; OMIM #227810), is an autosomal recessive disorder of glucose metabolism. It is also known as glycogen storage disease type XI. It is caused by mutation in the facilitative glucose transporter (*GLUT2*) gene that encodes the glucose transporter protein 2 expressed in hepatocytes, pancreatic beta cells, enterocytes, and renal tubular cells [1–3]. It is an extremely rare disorder first described in 1949 [4].

Approximately 109 cases worldwide were identified from 1949 to 2002, mainly from consanguineous communities [5]. None of the reported cases were from sub-Saharan Africa. Patients usually present early in life with rickets and hepatomegaly [6]. However, due to the variable presentation, the diagnosis of FBS is often missed or delayed. Other features of the syndrome include failure to thrive (FTT), fasting hypoglycemia, hyperglycemia, hypergalactosemia in the post-absorptive state, and hyperlipidemia [5, 7].

We report the case of a Ugandan boy who presented with failure to thrive (FTT) and rachitic features in infancy, but the diagnosis of FBS was made 3 years later due to limited diagnostic facilities and awareness regarding metabolic syndromes in our setting. Parental written informed consent and departmental approval was obtained to publish this case.

## Case presentation

This case report describes a 4-year-old boy born at term to non-consanguineous Black African parents by spontaneous vertex delivery with a birth weight of 3.0 kg. The pregnancy and perinatal period were uneventful.

He was referred from a rural hospital at the age of 11 months for further evaluation due to poor weight gain despite adequate breastfeeding. He was exclusively breastfed for 3 months and started formula feeds supplementation at 4 months without significant improvement in weight. There was no associated vomiting or diarrhea. He had multiple admissions for nutritional rehabilitation due to undernutrition with minimal weight gain. On weaning, he fed mainly on cow’s milk and hardly took any solid feeds from the family diet.

At approximately 1 year of age, he developed progressive abdominal distension despite low intake of feeds. There was no swelling in any other part of his body, no yellow discoloration of mucus membranes, and no seizures. He was HIV seronegative; there was no history of other chronic illnesses, and he was fully immunized. The child had delayed developmental milestones: social smile at 3 months, sitting without support at 12 months, and standing with support at 21 months. He is the seventh born child in a family of eight children. There is history of the death of two siblings. The first born was a boy delivered preterm who died at the age of 2 years 7 months with poor development and weight gain, and he had seizures at the time of death. The sixth born, a boy, with normal birth weight (3.2 kg) died at 1 year with a weight of 5 kg and he also had seizures. The other five siblings are alive and growing well.

**A physical examination** of this boy at 11 months revealed that he was small for age with a doll-like face; weight was 4.7 kg (weight for age *Z*-score − 5.41 SD); length was 62.5 cm (length for age *Z*-score − 4.47 SD); head circumference 42 cm (head circumference for age *Z*-score − 3.25 SD). He had widened anterior fontanelle of 4 cm × 3 cm, frontal bossing, widened wrists, three teeth (five to six expected), and rachitic rosary but no other bone deformities. His central nervous system revealed generalized hypotonia; an abdominal examination revealed a large liver of approximately 7 cm below the costal margin, which was smooth, firm, and nontender. No other abdominal masses were detected and other systemic examinations were normal.

**Fig. 1:**
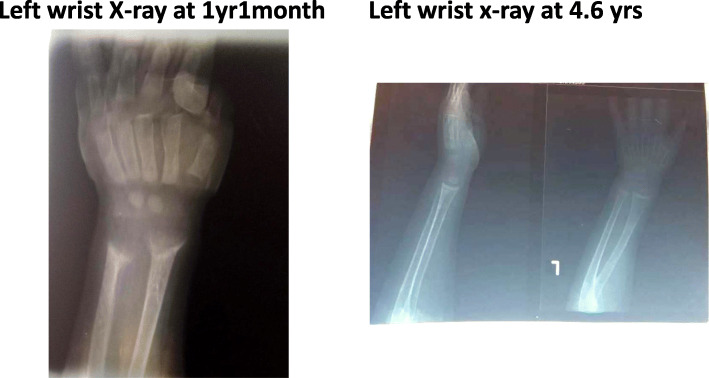
Left wrist X-rays

**Laboratory and radiological investigations** revealed low phosphorus of 0.58 mmol for which the normal range (NR) is 1.0–1.95 mmol, serum calcium 2.47 mmol (NR 2.25–2.75 mmol), markedly elevated alkaline phosphatase 1167 IU/L (NR up to 300 IU/L), normal serum albumin 3.7 g/dl, and vitamin D 22.3 ng/ml (NR above 30 ng/ml; after taking a high dose of 300,000 IU of parenteral vitamin D). His total white blood cell count was 15,000/ml, hemoglobin was 11.4 g/dl, platelets were 713,000/ml, and liver transaminases were normal. Initial X-rays of his left wrist revealed typical rachitic features of cupping and fraying at the wrist as shown in Fig. 1. An abdominal ultrasound revealed gross hepatomegaly, moderate splenomegaly, and normal echo texture of his liver. Other organs were normal; there was no evidence of nephrocalcinosis.

He was initially treated for vitamin D deficiency rickets with high dose of intramuscular 25(OH) vitamin D of 300,000 IU. However, after 2 months of treatment, there was no reported improvement in physical growth and development. The child was then managed as X-linked hypophosphatemic rickets due to persistently low phosphorus. He was initiated on orally administered phosphorus at a dose of 1 g/day in three divided doses and 1,25 OH vitamin D (calcitriol) 0.25 μg per day. There was reported improvement in development (he began to crawl and later walked) and he had some weight gain. However, at approximately 34 months of age, he developed massive abdominal distension with stagnation in weight. At 46 months of age, he was re-evaluated by the endocrinology team at Mulago National Referral Hospital, the teaching hospital for Makerere University, where further investigations were carried out. Fasting blood sugar was 2.2 mmol (NR 3.3–5.6) and after feeding it was 7.6 mmol, urine analysis showed pH 6.0 (NR 4.5–8), glucose 100 mg/dl (2+) (NR nil), ketones positive (2+) (NR nil), and protein was nil. Serum bicarbonate was 22.6 mmol (NR 22–29 mmol); sodium and potassium were normal. Fasting total cholesterol was 7.29 mmol (NR up to 5.2 mmol) and triglycerides 6.99 mmol (NR up to 1.7).

In view of the clinical features (doll-like facies, poor growth, hepatomegaly, and rachitic features) and laboratory results (hypophosphatemia, glycosuria, ketonuria in the presence of hypoglycemia, elevated cholesterol and triglycerides, and gross hepatomegaly on ultrasound scan) a diagnosis of FBS was made. No genetic testing was done to confirm the diagnosis. The child was initiated on a galactose-restricted diet; cornstarch was added to night meals, and sodium citrate was given at a dose of 15 mEq/kg per day in four divided doses. After 5 months of this therapy, his mother reported that he was more energetic and participated in more sustained play and had a weight gain of 0.8 kg. Table 1 summarizes key parameters on follow up.

**Table 1:** Summary of the clinical and laboratory investigations follow up

**Age (months)**	18	20	22	26	29	34	40	46	51	54	57
**Weight (kg)**	6.2	6.5	6.5	8.2	8.6	7.9	8.9	8.6	9.4	9.9	10.9
**Weight for age** ***Z*-score (SD)**	−4.84	−4.84	−4.91	−3.80	−3.70	−4.56	−4.17	−4.67	−4.37	−4.05	−3.60
**Length/height (cm)**	62.5	64	65			68.5	71	72	72.5	74.5	76.5
**Height for age** ***Z*-score (SD)**	−6.18	−6.04	−6.04			−6.97	−6.91	−7.05	−7.24	−7.22	−7.12
**Serum calcium (mmol) (NR 2.25–2.75)**		2.63	2.65	2.26	2.38	2.51	2.48	2.42	2.31	2.46	2.08
**Serum phosphorus (mmol) (NR 1.0–1.95)**	1.58	0.78	1.5	1.49	1.28	0.85	0.77	1.13	0.76	1.2	1.12
**Parathyroid hormone (pg/ml) (NR 15–65)**	169.9	62.27								55	
**Alkaline phosphatase (U/L) (NR up to 300 IU/L)**	539	417	403	376	637	499	307	240	319	434	400
**Bicarbonate (HCO**_**3**_**) (mmol) (NR 22–29)**								22.6	23.0		22.0
**Diagnosis**	Hypophosphatemic rickets	H	H	H	H	H	H	FBS	FBS	FBS	FBS
**Medication**	Calcitriol oral phosphorus	X	X	X	X	X	X	Calcitriol oral phosphorus HCO_3_	Y	Y	Y
**Dietary therapy**	Normal (N)	N	N	N	N	N	N	Galactose-restriction	G	G	G

*FBS* Fanconi–Bickel syndrome, *G* galactose-restricted diet, *H* hypophosphatemic rickets, HCO_3_ bicarbonate, *N* normal non-modified diet, *NR* normal range, *X* represents the medications calcitriol and orally administered phosphorus, *Y* represents medications calcitriol, orally administered phosphorus and orally administered bicarbonate

The mother’s perspective of the different therapies for our patient’s condition was that she saw marked improvement in his wellbeing on starting orally administered phosphorus and vitamin D; he was no longer getting ill with recurrent respiratory tract infections, and she noted marked progress in development although minimal weight gain. She believed that this could in fact be a case of hypophosphatemic rickets due to changes in the laboratory parameters for alkaline phosphatase and serum phosphorus on follow up. However, she became doubtful when she saw other children treated for hypophosphatemic rickets at the endocrinology clinic with a good catch up in growth. In addition, she was worried that the marked abdominal distension in her child could be another disease. On starting a galactose-free diet, she was worried that her child would not survive without milk because it is the child’s mainstay food. She was surprised that there was weight gain after stopping the milk and was later convinced to maintain the therapy.

## Discussion

FBS is an extremely rare metabolic disease characterized by hepatorenal glycogen accumulation, proximal renal tubular dysfunction, and impaired utilization of glucose and galactose [5, 7]. The common age of presentation is 2 months to 1 year.

Our patient presented at around 11 months with clinical features of proximal renal tubular dysfunction and florid rickets with hypophosphatemia, which was probably caused by phosphaturia due to proximal renal tubular dysfunction. In addition, we found glycosuria which would further confirm renal tubular dysfunction. However, we were unable to document phosphaturia, bicarbonaturia, and aminoaciduria due to the high cost of the tests. In addition, his typical doll-like facies, FTT, and abdominal distension made diagnosis of FBS highly probable. We believe that the abdominal distension was due to hepatic glycogen accumulation in his liver, as shown by the grossly enlarged liver on abdominal ultrasound scanning. Our patient also presented with fasting ketotic hypoglycemia which is commonly noted [8–10]. Genetic testing for confirmation was not done due to the unavailability of the appropriate test in our setting.

The clinical features of FBS are heterogeneous [11], just like the initial presentation of our patient; children may be misdiagnosed or treated for other isolated disorders of the syndrome, such as rickets [6], which may lead to delay in a definitive diagnosis. Furthermore, in limited-resource settings, the diagnosis may be missed due to limited investigative capacity and financial constraints. Missed or late diagnosis of FBS has been associated with higher rates of death related to liver failure and respiratory distress [2, 12, 13]. It is plausible that the diagnosis was missed in the two siblings of this child who died in early childhood. Their reported presentation of FTT is a characteristic feature of FBS.

This child was initially treated as having X-linked hypophosphatemic rickets because of the loss of two male siblings with almost similar clinical presentation; however, with detailed workup this was excluded. Other diagnoses like Fanconi syndrome were ruled out in this child due to the absence of typical proteinuria seen in Fanconi syndrome. In addition, the normal serum potassium and gross hepatomegaly were not in keeping with Fanconi syndrome. Von Gierke disease is an autosomal recessive disease of glycogen storage that is due to glucose-6-phosphatase deficiency leading to glycogen accumulation in the liver and kidneys. Features of Von Gierke disease include gross hepatomegaly, growth failure, and hypoglycemia with lactic acidosis and proteinuria. However, rickets are not a typical feature of Von Gierke disease.

FBS is an autosomal recessive disorder that is very rare and commoner in consanguineous societies. Consanguinity is not common among Black Africans. Although FBS has not been reported in any case reports describing Black Africans, poor response to therapy for hypophosphatemic rickets and worsening abdominal distension and FTT, raised our index of suspicion. There is no specific treatment available for this disorder. Treatment of rickets is done with vitamin D in the form of 1,25 dihydroxyvitamin D3 and phosphorus. We noted change in the attainment of developmental milestones in the child after initiation of phosphorus and vitamin D. It is also recommended to have citrate up to 15 mEq/kg per day to manage the acidosis and maintain bicarbonate more than 20 mEq/dL. Even though this patient had a bicarbonate level at a lower NR, we found it necessary to supplement him because of the proximal renal dysfunction that is reported in FBS. The renal tubular dysfunction causes loss of bicarbonate in urine that leads to acidosis. Acidosis contributes to the poor growth seen in FBS; therefore, supplementation with bicarbonate may act as a prophylaxis to avoid severe acidosis and subsequently improve growth. Cornstarch was added to our patient’s meals at night as he was found to have fasting hypoglycemia. Cornstarch provides glucose in slow release form and this maintains euglycemia during long periods between meals [14].

Galactose restriction is one of the mainstay therapies of children with FBS. Our patient’s main diet had contained cow’s milk for 4 years since weaning because he had poor appetite as reported by the mother. Milk contains a high content of galactose; we believe that if this diagnosis had been made earlier with a galactose-restricted diet, our patient’s outcome would have been much better with better physical growth and development. Early diagnosis and proper treatment in young children accelerate growth in height and weight and improve cognitive function. However, even in cases that are diagnosed late, proper dietary intervention should result in a reduction in the liver size and glycogen content [15] and avoidance of liver transplantation [16].

With early and appropriate treatment, the overall prognosis is very good, and survival to adulthood is favorable [10]. We are certain the prognosis of our patient is good and that we shall have catch up in growth for weight and height and development, as evidenced with the weight gain after initiation of therapy.

## Conclusion

In conclusion, the clinical, radiological, and biochemical parameters and response to therapy in this case are suggestive of FBS. This case report demonstrates that FBS can occur even in non-consanguineous Black African populations. Therefore, clinicians in resource-poor settings should raise their index of suspicion for such metabolic disorders in the setting of high prevalence of FTT among children.

## Data Availability

Not applicable.
